# Connective tissue growth factor promotes temozolomide resistance in glioblastoma through TGF-*β*1-dependent activation of Smad/ERK signaling

**DOI:** 10.1038/cddis.2017.248

**Published:** 2017-06-15

**Authors:** Huijun Zeng, Zhao Yang, Ningbo Xu, Boyang Liu, Zhao Fu, Changlin Lian, Hongbo Guo

**Affiliations:** 1 Department of Neurosurgery, The National Key Clinical Specialty, The Engineering Technology Research Center of Education Ministry of China, Guangdong Provincial Key Laboratory on Brain Function Repair and Regeneration, Zhujiang Hospital, Southern Medical University, Guangzhou 510282, China

## Abstract

Limited benefits and clinical utility of temozolomide (TMZ) for glioblastoma (GB) are frequently compromised by the development of acquired drug resistance. Overcoming TMZ resistance and uncovering the underlying mechanisms are challenges faced during GB chemotherapy. In this study, we reported that connective tissue growth factor (CTGF) was associated with GB chemoresistance and significantly upregulated in TMZ-treated GB cells. CTGF knockdown promoted TMZ-induced cell apoptosis and enhanced chemosensitivity, whereas its overexpression markedly conferred TMZ resistance *in vitro* and *in vivo*. Moreover, CTGF promoted TMZ resistance through stem-like properties acquisition and CD44 interference reversed the CTGF-induced TMZ resistance. Mechanistically, further investigation revealed that the TMZ-induced CTGF upregulation was tissue growth factor (TGF-*β*) dependent, and regulated by TGF-*β*1 activation through Smad and ERK1/2 signaling. Together, our results suggest a pivotal role of CTGF-mediated TMZ resistance through TGF-*β*1-dependent activation of Smad/ERK signaling pathways. These data provide us insights for identifying potential targets that are beneficial for overcoming TMZ resistance in GB.

Glioblastoma (GB) represents the most prevalent type and lethal primary brain tumor in adults, with high aggressive progression, weak response to cancer therapy and bad prognosis.^1^ Currently, the standard treatment of GB is multimodal involving maximal surgical resection followed by concurrent radiation and chemotherapy with alkylating drugs.^2^ Though these treatment regimens have been shown to prolong survival, the poor median survival time with ~14.6 months points to urgent need for new chemotherapeutic approaches.^3^ Temozolomide (TMZ), as first-line therapy for GB treatment, is frequently limited in durability of treatment response by chemoresistance. Therefore, identifying novel mechanisms of TMZ resistance could provide potential molecular targets for GB therapy.

Connective tissue growth factor (CTGF) is a secreted protein that belongs to the CCN family, which consists of the following six members: cysteine-rich protein 61 (CYR61/CCN1), CTGF (CCN2), nephroblastoma overexpressed gene (NOV/CCN3), Wnt-inducible secreted protein-1 (WISP-1/CCN4), WISP-2 (CCN5) and WISP-3 (CCN6),^4^ and acts as a multifunctional signaling modulator in various biological or pathological processes.^5^ To date, about 27 human cancers have been linked to the aberrant CTGF expression, and the role of deregulated CTGF is complicated with tissue-dependent manner and leads to varying survival outcomes.^6^ Previous studies have reported that CTGF overexpression promoted tumor development and progression,^7^ and enhanced cancer cell migration and metastasis.^8, 9^ Moreover, CTGF expression is associated with chemoresistance in glioma, breast cancer, osteosarcoma and thyroid cancer.^10, 11, 12, 13^ In glioma, CTGF conferred resistance to chemotherapeutic agents through anti-apoptotic survivin and FLIP expression, or inhibition of pro-apoptotic PARP expression.^10^ However, molecules and signaling pathways regulating CTGF expression in TMZ resistance are still elusive.

Tissue growth factor-*β* (TGF-*β*), a profibrotic cytokine as well as a growth factor involved in multiple pathophysiological processes, is one of the most prominent factors regulating CTGF expression in many cells.^14, 15, 16^ The molecular details how TGF-*β* induces CTGF expression are still largely unsolved. Leask *et al.*^17^ suggested an ALK5-dependent mechanism as dominant for this induction and other signaling pathways such as the TGF-*β* non-canonical MAPK, PI3K/Akt pathways may also participate in this process. Recently, TGF-*β* is recognized as an emerging player in drug resistance,^18, 19^ which correlated with the epithelial-to-mesenchymal transition (EMT) and cancer stem cells (CSCs) phenotype modulations.^20^ Additionally, evidence also revealed that chemotherapy treatment itself induced EMT process or mesenchymal phenotypes.^21, 22^ However, whether TGF-*β* signaling is activated and involves in chemotherapy-induced drug resistance is largely unknown.

In this study, we showed that CTGF expression is upregulated during TMZ treatment and conferred TMZ resistance through the acquisition of stem-like properties in GB cells. Moreover, the TGF-*β* signal was activated during this process and acted as an essential upstream signaling molecule in CTGF mediation via Smad and ERK1/2 signal pathways. Targeting TGF-*β*/CTGF axis may provide novel insights for management of TMZ resistance in GB chemotherapy.

## Results

### CTGF expression associates with glioma grades and chemoresistance

**Figure 1:**
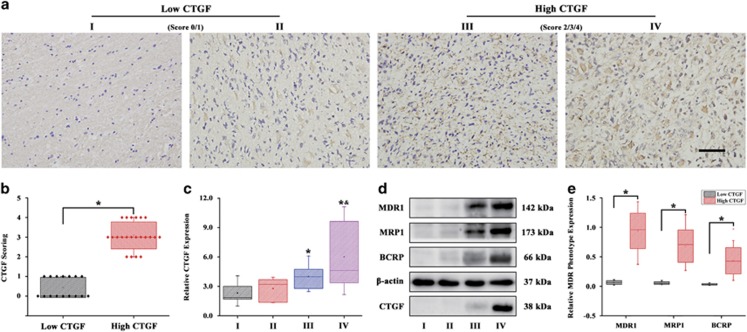
CTGF expression associates with GB grades and chemoresistance. The immunohistochemical staining of CTGF expression in GB tissues with different tumor grades (**a**) and the CTGF scoring analysis (**b**), I (*n*=7); II (*n*=9); III (*n*=6); IV (*n*=16), brown staining indicated the positive immunoreactivity, the tumor grade I and II with low CTGF staining was set as the low CTGF group (score 0/1), while grade III and IV with positive CTGF staining was set as the high CTGF group (score 2/3/4), scale bar=100 *μ*m, *P<0.05. The qPCR analysis of CTGF expression in GB tissues with different tumor grades (**c**), *P<0.05 compared to the GB grade I; ^&^*P*<0.05 compared to the GB grade II. Protein expression of multidrug resistance phenotype (MDR1, MRP1, BCRP1) and CTGF in different tumor grades of GB by western blot analysis (**d**) and further protein band density quantification analysis (**e**); *β*-actin served as the loading control, *P<0.05. Results above are expressed as the mean±S.D. of three independent experiments

Recent studies have demonstrated that CTGF could confer resistance to chemotherapeutic agents in a variety of cancers.^10, 11, 12, 13^ Moreover, in our previous microarray analysis, we also found CTGF upregulated in the TMZ-resistant U87 cells.^23^ However, whether the deregulated CTGF expression correlated to the glioma tumor grades and the tumor grades-related multidrug resistance (MDR) phenotype are still not clear. Here, we hypothesized that CTGF may involve in the glioma chemoresistance during TMZ treatment. First, surgical resection specimens from 38 glioma patients who are receiving neoadjuvant chemotherapy were analyzed for the correlations between CTGF expression and glioma grades through immunohistochemical (IHC) staining and qPCR analysis. IHC staining indicated that patients with high level of positive CTGF expression corresponded to the high tumor grade (Figure 1a) and further confirmed by the IHC score analysis (Figure 1b). In addition, the qPCR analysis showed higher tumor grade with elevated CTGF expression, especially for glioma grade III and IV (Figure 1c). Then we examined the relationship between CTGF expression and drug resistance in glioma. Western blot analysis revealed that expressions of MDR phenotype (MDR1, MRP1, BCRP1) was increased with the elevated tumor grades, and more interestingly, with CTGF upregulation (Figure 1d). To further demonstrate whether the MDR phenotype correlated to the CTGF protein expression, the protein band density quantification analysis of the western blot upon 38 glioma tissue samples was applied. The results showed that the high CTGF group (grade III and IV) had a significantly higher MDR phenotype expression than the low CTGF group (grade I and II) (Figure 1e). These data suggested that CTGF expression was associated with glioma tumor grades and chemoresistance.

### CTGF is markedly upregulated in GB cells after TMZ treatment

**Figure 2:**
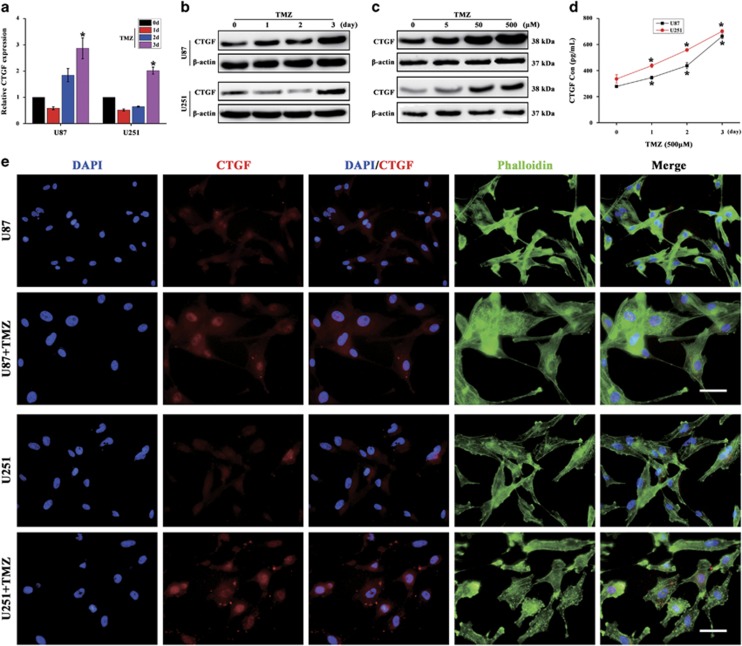
TMZ treatment increases CTGF expression in GB cells. Relative CTGF expression of TMZ-treated (500 *μ*M) U87 and U251 cells during 3 days by qPCR (**a**) and western blot analysis (**b**), *P<0.05 compared to the cells on day 0. U87 and U251 cells were treated with different TMZ concentrations (0, 5, 50, 500 *μ*M) for 3 days and CTGF protein expression was analyzed by western blot, and *β*-actin served as the loading control (**c**). U87 and U251 cells were treated with 500 *μ*M TMZ for 3 days and the culture supernatants were collected for CTGF expression by ELISA assay, *P<0.05 compared to non-treated cells (**d**). Immunofluorescent analysis of CTGF expression in TMZ-treated GB cells for 3 days, scale bar=50 *μ*m (**e**). Results above are expressed as the mean±S.D. of three independent experiments

To investigate CTGF expression during GB cells chemotherapy, we treated U87 and U251 cells with TMZ at different concentrations and time points. First, U87 and U251 cells were treated with different TMZ concentrations for 24 h and determined their half maximal inhibitory concentration (IC_50_), respectively. Survival assays showed that the IC50 of U87 and U251 cells is about 1.0 mM and further confirmed by the Live & Dead staining (Data not shown). We thus decided to evaluate the implication of TMZ concentration within 500 *μ*M (1/2 IC_50_) on CTGF expression in GB cells. Results revealed that TMZ at 500 *μ*M concentration significantly increased the CTGF mRNA and protein expression, especially on day 3 (Figures 2a and b). In addition, to explore the effect of TMZ concentrations upon the CTGF expression, GB cells were treated with TMZ at different concentrations (0, 5, 50, 500 *μ*M) for 3 days and immunoblot assays showed that CTGF expression was upregulated with increasing TMZ concentrations (Figure 2c). These data suggested TMZ could induce CTGF expression with time and concentration-dependent manner in GB cells.

Since CTGF is a secreted protein, enzyme-linked immunosorbent assay (ELISA) was applied to detect the CTGF expression in culture medium and immunofluorescence was used to examine its cellular localization after 3 day TMZ exposure in GB cells. Following TMZ treatment, we found the total CTGF protein expression in TMZ-treated cell culture medium was time dependently increased within 3 days by ELISA (Figure 2d). We also observed morphological changes (Cytoskeleton, green), for example, larger, irregular morphology and long protrusions in both TMZ-treated cells, and relative higher CTGF expression was detected within nuclear or cytoplasm in TMZ-treated cell groups (Figure 2e). These results indicated that TMZ could induce CTGF expression in GB cells during TMZ treatment *in vitro*.

### CTGF confers resistance to TMZ in GB cells

**Figure 3:**
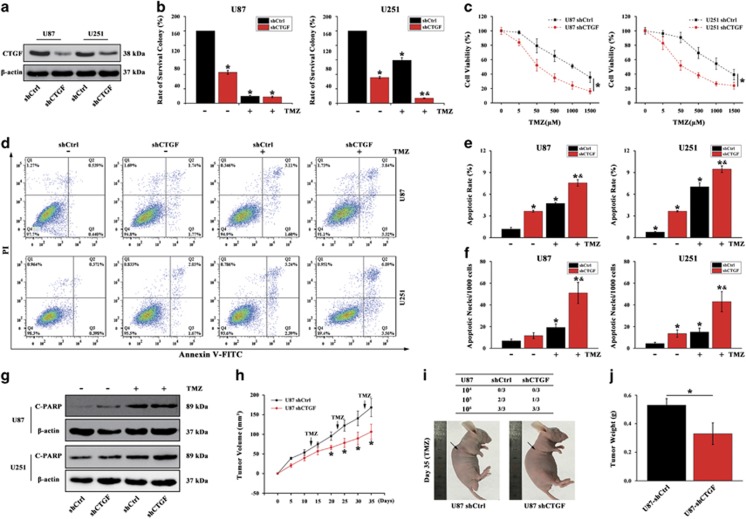
CTGF depletion enhances TMZ chemosensitivity to GB cells. Western blot analysis of CTGF expression in U87 and U251 cells transfected with shCTGF or shCtrl, respectively. *β*-actin served as the loading control (**a**). Colony forming ability of CTGF transfected or non-transfected U87 and U251 cells in the absence or presence of TMZ by Giemsa staining, *P<0.05 compared to non-treated shCtrl cells, ^&^*P*<0.05 compared to TMZ-treated shCtrl cells (**b**). Cell viability of U87 and U251 shCTGF or shCtrl cells treated with different TMZ concentrations for 24 h and determined by CCK-8 assay. Data were showed as percentage of cells treated without TMZ, *P<0.05 compared to non-treated shCtrl cells (**c**). U87 and U251 shCTGF or shCtrl cells were exposed to 500 *μ*M TMZ for 48 h and the apoptosis was analyzed by flow cytometry (**d**, **e**), Hochest 33342 staining (**f**) and cleaved PARP protein expression (**g**); *β*-actin served as the loading control, *P<0.05 compared to shCtrl cells treated without TMZ, ^&^*P*<0.05 compared to shCtrl cells with TMZ treatment. Growth curve of U87 shCTGF or shCtrl cells-derived subcutaneous tumor xenografts after TMZ treatment, *P<0.05 compared to the shCtrl cells-derived tumor xenograft (**h**). Representative images of tumors originated from U87 shCtrl or shCTGF cells on day 35 (**i**) and mean of tumor weight (**j**), *P<0.05. Results above are expressed as the mean±S.D. of three independent experiments

To determine whether CTGF is associated with TMZ resistance *in vitro*, GB cells were transfected with CTGF or Control shRNA. The knockdown efficiency was confirmed by western blot (Figure 3a). Through Giemsa staining, we found that loss of CTGF in GB cells significantly decreased colony forming ability upon exposure to TMZ (Figure 3b) and dramatically enhanced TMZ chemosensitivity toward the GB cells (Figure 3c). We also assessed the apoptotic rate in CTGF knockdown or shCtrl cells in response to TMZ determined by flow cytometry (Figure 3d). Through data analysis, we found only (4.74±0.14)% of U87 shCtrl and (7.03±0.54)% of U251 shCtrl cells that underwent either apoptosis or death compared with (7.58±0.45)% and (9.48±0.46)% of the U87 shCTGF and U251 shCTGF cells after exposure to TMZ (500 *μ*M) 48 h, respectively (Figure 3e). Similarly, Hoechst 33342 staining showed that the percentage of apoptotic cells was also higher in TMZ-treated U87 shCTGF and U251 shCTGF cells than that of U87 shCtrl and U251 shCtrl cells (Figure 3f). Apoptosis was further verified by cleaved PARP expression and the results showed that TMZ treatment caused robust cleaved PARP expression in U87 shCTGF and U251 shCTGF cells as compared with that of U87 shCtrl and U251 shCtrl cells (Figure 3g). To examine whether CTGF knockdown correlated to the TMZ resistance *in vivo*, U87 shCtrl or shCTGF cells were subcutaneously injected into the immunocompromised nude mice. After the tumor volume reached 50 mm^3^, the tumor-bearing mice were treated with TMZ (50 mg/kg/day) for 5 days per week for three cycles. Results showed that CTGF knockdown significantly inhibited the tumor growth with decrease in tumor volume and weight upon TMZ treatment as compared to the group of U87 shCtrl cells injection (Figures 3h–i). These above data confirmed that CTGF depletion significantly enhanced the TMZ chemosensitivity and inhibited the tumor growth in glioma.

**Figure 4:**
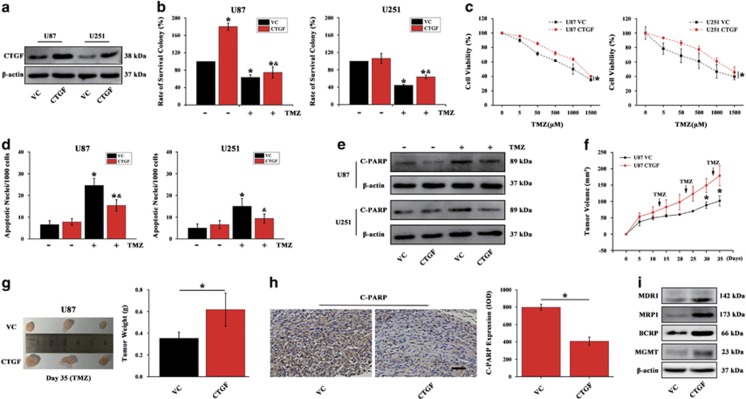
CTGF overexpression confers resistance to TMZ. Western blot analysis of CTGF expression in U87 and U251 cells transfected with CTGF or Vector Control (VC), respectively. *β*-actin served as the loading control (**a**). Colony forming ability of U87 and U251 CTGF overexpressing or VC cells in the absence or presence of TMZ by Giemsa staining, *P<0.05 compared to non-treated VC cells, ^&^*P*<0.05 compared to TMZ-treated VC cells (**b**). Cell viability of U87 and U251 CTGF or VC cells treated with different TMZ concentrations for 24 h and determined by CCK-8 assay. Data were showed as the percentage of cells treated without TMZ (**c**). U87 and U251 CTGF or VC cells were exposed to 500 *μ*M TMZ for 48 h and the apoptosis was measured by Hoechst 33342 staining (**d**) and cleaved PARP protein expression (**e**), *P<0.05 compared to VC cells treated without TMZ, ^&^*P*<0.05 compared to VC cells with TMZ treatment. Growth curve of U87 CTGF or VC cells-derived subcutaneous tumor xenografts after treatment with TMZ, *P<0.05 compared to VC cells-derived tumor xenograft (**f**). Representative images of tumors originated from U87 CTGF or VC cells on day 35 and mean of tumor weight, *P<0.05 (**g**). Immunohistochemistry and relative cleaved PARP expression analysis in U87 CTGF or VC cells-derived tumor xenograft, scale bar=200 *μ*m, *P<0.05 (**h**). Western blot analysis of MDR1, MRP1, BCRP1, MGMT protein expressions in U87 CTGF or VC cells-derived tumor xenograft (**i**). Results above are expressed as the mean±S.D. of three independent experiments

If CTGF depletion could enhance TMZ chemosensitivity in glioma, we asked whether CTGF overexpression could confer resistance to TMZ. Lentiviral activation particles were used for CTGF overexpression in U87 and U251 cells, and confirmed by western blot (Figure 4a). Results showed CTGF overexpression could significantly rescue the reduction of colony forming capacity (Figure 4b) and decrease the TMZ-induced cell death in U87 CTGF and U251 CTGF cells (Figure 4c). Furthermore, CTGF overexpression could decrease the TMZ-induced cell apoptosis revealed by Hoechst 33342 staining (Figure 4d) and inhibit cleaved PARP protein expression (Figure 4e). Additionally, *in vivo* study showed that the mice injected with U87 CTGF cells had a significant increase in tumor volume and weight upon TMZ treatment as compared to that of U87 VC cells injection (Figures 4f and g). We also performed IHC analysis to verify cleaved PARP expression. In U87 VC cells-derived tumor xenograft, we observed significantly increased cleaved PARP expression upon TMZ treatment in comparison with the U87 CTGF xenografts (Figure 4h). Moreover, xenografts derived from U87 CTGF cells showed higher expression of MDR phenotype when compared to that of U87 VC cells (Figure 4i). Together, these data suggested that inhibition of CTGF sensitizes GB cells to TMZ and its overexpression confers TMZ resistance *in vitro* and *in vivo*.

### CTGF-mediated TMZ resistance is through stem-like properties acquisition

**Figure 5:**
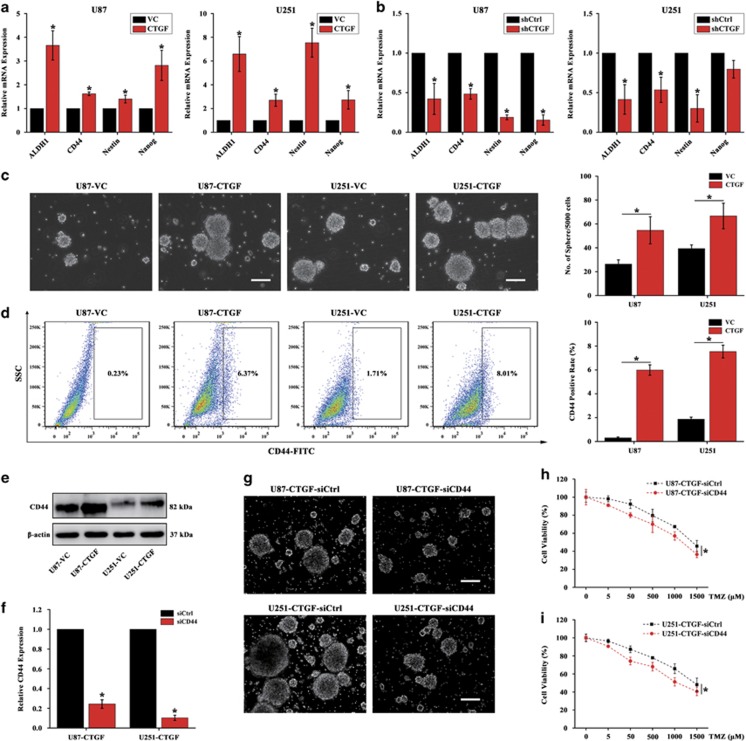
Stem-like properties acquisition involves in CTGF-mediated TMZ resistance. qPCR analysis of stemness factors (ALDH1, CD44, Nestin, Nanog) expression in U87 or U251 CTGF overexpression (**a**) and knockdown (**b**) cells, *P<0.05. Representative images of tumorsphere originated from U87 and U251 CTGF or VC cells cultured in Complete MammoCult Medium for 7 days and the quantitative analysis of tumorspheres (**c**), scale bar=50 *μ*m, *P<0.05 as compared to VC cells. The proportion of CD44^+^ cells in U87 and U251 CTGF or VC cells cultured in Complete MammoCult Medium detected by flow cytometry and further CD44^+^ rate analysis (**d**), *P<0.05 as compared to the VC cells. CD44 expression in U87 and U251 CTGF or VC cells detected by western blot analysis (**e**), *β*-actin served as the loading control. CD44 siRNA was transfected into the stable U87 and U251 CTGF cells, and analyzed by qPCR (**f**), *P<0.05. Representative images of tumorsphere formation in co-transfected CTGF-siCD44 or siCtrl U87 and U251 cells (**g**), scale bar=50 *μ*m. The effect of siCD44 on cell viability in U87 and U251 CTGF cells treated by different TMZ concentrations for 24 h (**h**, **i**), *P<0.05 as compared to CTGF-siCtrl cells. Results above are expressed as the mean±S.D. of three independent experiments

Accumulating evidence has showed that chemotherapy failure might be blamed for the existence of CSCs,^24, 25, 26, 27^ and we speculated that CTGF may mediate TMZ resistance through stem-like properties acquisition. To examine this notion, we evaluated the stem-like properties expression in CTGF overexpressed or knockdown GB cells. Through qPCR analysis, we found ectopic expression of CTGF increased the stemness factor ALDH1,^28^ CD44,^29^ Nestin and Nanog expressions (Figure 5a), whereas CTGF knockdown dramatically decreased its stemness expression (Figure 5b). Furthermore, U87 and U251 CTGF or VC cells were then subjected to tumorsphere forming assay with serum-free culture for 7 days. Results showed that the cells with ectopic CTGF expression significantly enhanced the tumorsphere forming ability (Figure 5c) and increased the proportion of CD44^+^ cells (Figure 5d), which was further confirmed by western blot (Figure 5e). Since CD44 was one of the important surface marker on CSCs,^30^ we next sought to determine whether CD44 repression could alleviate CTGF-induced TMZ resistance. The CD44 short interfering RNA (siRNA) was applied and transfected into the stable U87 CTGF and U251 CTGF cells (Figure 5f). The tumorsphere forming assay showed that CD44 interference significantly decreased tumorsphere forming ability in CTGF overexpression GB cells as compared to that of the siCtrl group (Figure 5g). Furthermore, we found that with CD44 repression cells chemosensitized to TMZ in U87 CTGF and U251 CTGF cell group, while with relative higher cell viability upon TMZ treatment in the siCtrl cell group (Figures 5h and i). Taken together, these results suggested that CTGF promote TMZ resistance through stem-like properties acquisition, and stemness mediation may reverse CTGF-induced TMZ resistance.

### TGF-*β* signal activation is required for the TMZ-induced CTGF upregulation

**Figure 6:**
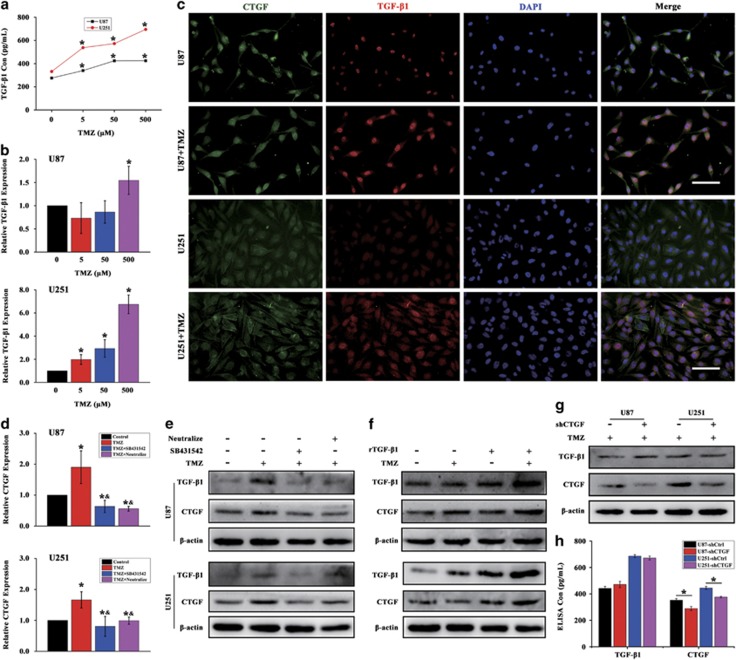
TGF-*β* signal activation is required for the TMZ-induced CTGF upregulation. U87 and U251 cells were treated with different TMZ concentrations for 24 h and the culture supernatants were collected for TGF-*β*1 expression by ELISA assay, *P<0.05 compared to non-treated cells (**a**). qPCR analysis of TGF-*β*1 expression in U87 and U251 cells treated with or without different TMZ concentrations for 24 h, *P<0.05 compared to non-treated cells (**b**). Immunofluorescence analysis of CTGF and TGF-*β*1 expression with or without TMZ treatment in U87 and U251 cells, scale bar=100 *μ*m (**c**). U87 and U251 cells were, respectively, treated with TGF-*β* receptor inhibitor SB431542 or TGF-*β*1/2/3 neutralizing antibody for 24 h, and analyzed by qPCR (**d**) and western blot analysis (**e**). *P<0.05 compared to non-treated cells, ^&^*P*<0.05 compared to cells treated with TMZ alone. U87 and U251 cells were treated with recombinant TGF-*β*1 and/or TMZ for 24 h and analyzed for TGF-*β*1 and CTGF expression by western blot (**f**). The GB shCTGF and shCtrl cells were treated with TMZ for 24 h, western blot (**g**) and ELISA (**h**) analysis were performed for TGF-*β*1 and CTGF expression, *β*-actin served as the loading control, *P<0.05 compared to the shCtrl cells. Results above are expressed as the mean±S.D. of three independent experiments

Previous studies have demonstrated the secreted matricellular protein CTGF could interact with multiple molecules and participate in various regulation of important cell functions.^31, 32, 33^ Among these multiple molecules, TGF-*β* was reported to act upstream and synergize with action of the CTGF in cellular functions.^14, 15, 16^ To this end, we hypothesized whether TGF-*β* signal was activated in the process of TMZ-induced CTGF upregulation during GB chemotherapy. First, we treated U87 and U251 cells with different TMZ concentrations and determined the TGF-*β*1 protein expression in the culture supernatants. Through ELISA, we found that total TGF-*β*1 secretion was concentration dependently increased between 5 and 500 *μ*M, and relative higher TGF-*β*1 protein expression was induced in U251-treated cells (Figure 6a). Next, we reasoned the enhanced TGF-*β*1 secretion was the result of enhanced transcriptional activity of TGF-*β*1 and assessed its mRNA expression by qPCR. As shown in Figure 6b, the TGF-*β*1 expression was increased with the elevated concentrations, especially in U251-treated cells, which was consistent to the results of ELISA assay. Given the similar profile of TMZ-induced CTGF and TGF-*β*1 expression, we sought to study whether TGF-*β*1 and CTGF has co-expression profiling after TMZ treatment. Immunofluorescence showed that the elevated TMZ-induced CTGF expression (green) in GB cells was accompanied with increased TGF-*β*1 expression (red), and both the TGF-*β*1 and CTGF expression extended to cytoplasm as compared to the Control group after 24 h TMZ treatment (Figure 6c). These data suggested that TMZ-induced CTGF upregulation may associate with the TGF-*β*1 signal activation in GB cells *in vitro*.

To confirm whether TGF-*β*1 signal activation is required for TMZ-induced CTGF upregulation, a potent and specific TGF-*β*1 type I receptor (T*β*RI) inhibitor SB431542 and anti-TGF-*β*1/2/3 neutralizing antibody were used. qPCR analysis showed that TGF-*β*1 inhibition by SB431542 and neutralizing antibody significantly decreased the CTGF mRNA expression as compared to the cells treated with TMZ alone, (Figure 6d) and this was further confirmed by western blot analysis (Figure 6e). Additionally, exogenous recombinant human TGF-*β*1 (rTGF-*β*1) was also applied in this study. Western blot analysis showed that rTGF-*β*1 increased the CTGF expression and with significant upregulation of TGF-*β*1 and CTGF expression in cells treated by rTGF-*β*1 and TMZ as compared to cells treated with TMZ alone (Figure 6f). Moreover, to explore whether there is an amplification loop between CTGF and TGF-*β*1 expression, western blot and ELISA analysis were applied to assess the CTGF and TGF-*β*1 expression in GB shCTGF cells during TMZ treatment. In Figure 6g, we observed significant decreased CTGF expression in CTGF downregulated GB cells while with no difference in TGF-*β*1 expression as compared with the shCtrl group, which further confirmed by the ELISA analysis (Figure 6h). Therefore, the upstream TGF-*β* signal could regulate CTGF expression and its activation is required for the CTGF upregulation during TMZ treatment.

### Smad and ERK pathways are involved in TMZ-induced TGF-*β*1/CTGF signaling activation

**Figure 7:**
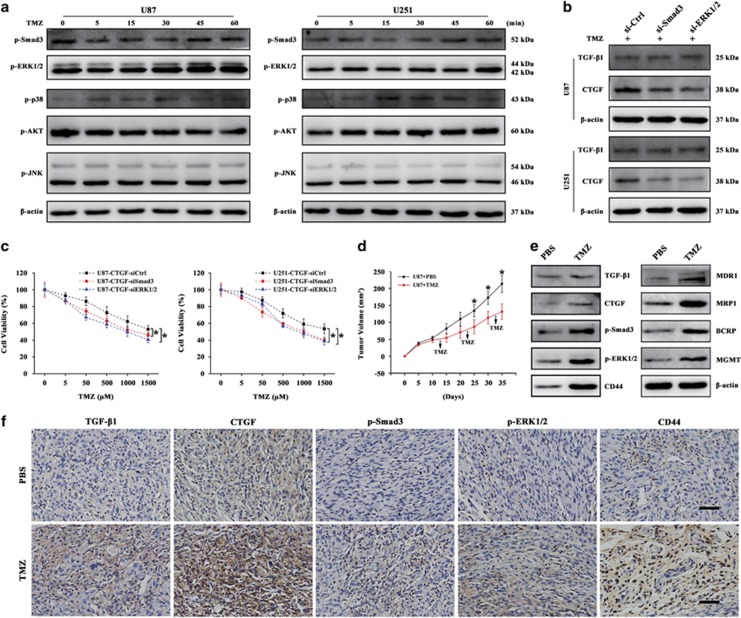
Smad and ERK pathways are involved in TMZ-induced TGF-*β*1/CTGF signaling activation. U87 and U251 cells were treated by TMZ within 60 min and subjected to western blot analysis for p-Smad3, p-ERK1/2, p-p38 MAPK, p-AKT, p-JNK protein expressions (**a**). TGF-*β*1 and CTGF protein expression after Smad3 or ERK1/2 interference during TMZ treatment in U87 and U251 cells (**b**). Effect of siSmad3 or siERK1/2 on cell viability in CTGF overexpression U87 and U251 cells treated by different TMZ concentrations, *P<0.05 compared to the CTGF-siCtrl cells (**c**). Growth curve of U87 cells-derived subcutaneous tumor xenografts with or without TMZ treatment, *P<0.05 compared to the TMZ-treated tumor xenograft group (**d**). The protein expression of TGF-*β*1/CTGF signal axis and multidrug resistance phenotype (MDR1, MRP1, BCRP) and MGMT in *in vivo* xenograft tumor treated by TMZ or PBS on day 35 (**e**), *β*-actin served as the loading control. Immunohistochemical staining of TGF-*β*1, CTGF, p-Smad3, p-ERK1/2 and CD44 in xenograft tumor specimens. Brown staining indicates positive immunoreactivity, scale bar=100 *μ*m (**f**). Results above are expressed as the mean±S.D. of three independent experiments

The TGF-*β* signal exerts its functions by activating canonical or non-canonical signaling pathways. In the canonical pathway, the downstream signaling activates upon Smad2/3 phosphorylation,^34^ while the non-canonical signaling pathway, which is also called the non-Smad signal pathway, is involved the activation of ERK, p38 MAPK, PI3K/Akt and JNK signaling pathways.^35^ We therefore analyzed the phosphorylation of Smad3, ERK1/2, p38 MAPK, Akt and JNK signals after TMZ treatment within 60 min. Among these molecular pathways, we found the Smad and ERK1/2 pathways were activated with increased phosphorylation after TMZ treatment (Figure 7a). To further determine whether TGF-*β* regulates CTGF expression through the Smad and ERK1/2 signal transduction, Smad3 and ERK1/2 siRNA were used during TMZ treatment. As shown in Figure 7b, the siRNA-mediated Smad3 or ERK1/2 blockade could downregulate the TMZ-induced CTGF expression as compared to the siCtrl group. Meanwhile, both blockades did not affect the TMZ-induced TGF-*β*1 expression. This suggested that the Smad or ERK1/2 signal blocking could disturb the TGF-*β*1 downstream CTGF signaling activation during TMZ treatment. In addition, we also found Smad3 or ERK1/2 blockade promoted TMZ-induced cell death and rescued CTGF-medicated TMZ resistance in U87 and U251 CTGF cells (Figure 7c). Therefore, Smad and ERK1/2 pathways are involved in TMZ-induced TGF-*β*1/CTGF signaling activation during TMZ treatment.

To confirm whether long-term TMZ therapy could induce chemoresistance and the TGF-*β*1/CTGF signal axis was activated during this process *in vivo*, we preformed tumor xenograft model using subcutaneously injection of U87 cells and treated with or without TMZ during tumor formation. As shown in Figure 7d, TMZ significantly decreased the U87 cells-derived tumor formation as compared to the PBS Control group. Meanwhile, western blot analysis revealed that xenograft tumor treated by TMZ showed increased expression of TGF-*β*1/CTGF signal axis and higher expression of MDR phenotype (MDR1, MRP1, BCRP) and MGMT on day 35 as compared to the PBS Control group (Figure 7e). This indicated that with long-term TMZ therapy, the GB xenograft tumor acquired drug-resistant phenotype and became chemoresistant to TMZ therapy. Next, the expression of TGF-*β*1/CTGF signal axis was confirmed by IHC analysis. Relatively higher expressions of TGF-*β*1, CTGF, p-Smad3, p-ERK1/2 and CD44 protein were observed in xenograft tumor treated with TMZ (Figure 7f). Together, these data validated that TMZ treatment could induce chemoresistance through TGF-*β*1/CTGF signal axis along with Smad and ERK signal activation *in vivo*.

## Discussion

Acquired chmoresistance to alkylating agents is a major cause of treatment failure in GB patients. Developing effective strategies to overcome chemoresistance for GB represents urgent clinical need. TMZ, as frontline chemotherapeutic agent for GB, is frequently limited in durability of treatment response by drug resistance development. Though diverse mechanisms toward TMZ resistance, for example, DNA O^6^-methylguanine methyltransferase (MGMT), DNA mismatch repair (MMR), base excision repair (BER), ATP-binding cassette (ABC) protein family, have been demonstrated so far,^36, 37, 38, 39, 40^ more effort is still needed for identifying novel mechanisms underlying acquired TMZ resistance. Herein, we show that TMZ treatment could activate the TGF-*β*/CTGF axis pathway with Smad and ERK1/2 signal-dependent manner that contribute to the acquired TMZ resistance. Targeting TGF-*β*/CTGF signal axis may provide new strategies for overcoming TMZ resistance in GB.

Previous studies showed that CTGF expression had a significant correlation with GB patient survival and suggested CTGF may have prognostic significance.^41^ Approximately 44% of human cancers with deregulated CTGF expression depend on the degrees of tumor differentiation.^6^ Moreover, CTGF was reported to correlate with chemotherapeutic resistance in a variety of cancers, and CTGF could promote cell progression through supporting tumor cell survival and drug resistance.^10, 11, 12, 13^ However, whether the deregulated CTGF expression correlated to the GB tumor grades and the MDR phenotype expression depend on tumor grades are still not clear. In this study, we found CTGF expression positively correlated to the GB grades and with relative higher CTGF expression in GB grade III and IV. These results indicated that CTGF associates with the tumor grades and correlates to drug resistance phenotype in GB.

Upon CTGF functional analysis, we found that CTGF depletion decreased colony forming ability, enhanced TMZ chemosensitivity to GB cells and promoted cell apoptosis, and further CTGF overexpression conferred resistance to TMZ. The role of CTGF in chemoresistance was reported in various cancers and mainly shown to associate with anti-apoptotic proteins, for example, Bcl-xL, cIAP1, Survivin, Flip or pro-apoptotic protein PARP.^10, 11, 12^ In our study, we confirmed that CTGF-mediated TMZ resistance was associated with pro-apoptotic protein cleaved PARP expression, and these results suggested that apoptosis modulation was one important mechanism in CTGF-mediated TMZ resistance in GB. In Chang *et al.*^42^ study, they demonstrated that CTGF expression could activate pluripotency genes *NANOG*, *SOX2* and *POU5F1* in head and neck cancer cells, and promote mesenchymal-epithelial transition, the reverse process of EMT. Herein, we found CTGF overexpression increased stemness-related gene expressions, while CTGF knockdown caused a decrease in these genes expressions. Further analysis using CD44 siRNA revealed that stemness interference could alleviate CTGF-induced drug resistance. From this perspective, the specific targeting of CSCs, together with conventional chemotherapy or radiotherapy, may achieve stable remission or cure cancer.^43, 44^ Auffinger *et al.*^45^ showed that therapeutic doses of TMZ consistently increased the glioma stem cells (GSC) pool both *in vitro* and *in vivo*, and provided the first evidence that chemotherapeutic agents can able to interconvert between non-GSCs and GSCs. Recent study of their group revealed this conversion of glioma cells dedifferentiation to glioma stem-like cells was attributed to the therapeutic TMZ stress-induced HIF signaling.^46^ Therefore, the role of TMZ dedifferentiation may involve in this process and the underlying mechanisms still needed more investigations. Here, we initially identified CTGF expression conferred resistance to TMZ through stemness acquisition during TMZ chemotherapy.

CTGF is a secreted matricellular protein that participates in various important cell function regulations, and modulates a variety of signaling pathways through interaction with cytokines and growth factors including the TGF-*β* signal.^15, 47^ The TGF-*β* signal was reported as a strong inducer and acted upstream of CTGF, which contains a unique TGF-*β* inducible element on its promoter.^14, 15, 16^ Our finding showed that TGF-*β*1 protein and transcriptional expression were upregulated during TMZ treatment, and this elevated TGF-*β* expression may associate with cell response to chemotherapy.^48^ Next, we confirmed that TGF-*β* signal inhibition could inhibit the CTGF expression through the T*β*RI inhibitor SB431542 and neutralizing antibody, while on the other side, the addition of rTGF-*β*1 promoted CTGF expression and with significant TGF-*β*1 and CTGF upregulation in rTGF-*β*1, and TMZ combined treatment. Therefore, we preliminary confirmed that, to some extent, TMZ-induced TGF-*β*1 expression contributes to the CTGF upregulation, and TGF-*β*1 activation is required for CTGF expression.

It has been reported that CTGF enhances the binding of TGF-*β*1 to its cognate receptors and activates Smad and non-Smad signaling.^16, 33^ In this study, the Smad and ERK signaling were both activated in TMZ-treated GB cells, and the interference of Smad3 and ERK1/2 signal by siRNA decreased the TMZ-induced CTGF expression. This indicated that TGF-*β*1 mediated CTGF expression through multiple pathways, which may be the reason for the inconsistent results showed in TGF-*β*1 activation assay. What’s more, Smad3 and ERK1/2 blockade chemosensitized the CTGF overexpressed GB cells to TMZ. However, the distinct function between Smad3 and ERK1/2 pathways involved in this study remains unclear, and these may be our future research direction. In addition, we further validated that the TGF-*β*1/CTGF signal axis was activated *in vivo* during TMZ treatment, and these findings may shed light on the clinical significance of these molecular pathways in preventing drug resistance during GB chemotherapy. Collectively, our *in vitro* and *in vivo* data suggest that CTGF is critical in the mediation of TMZ resistance in GB through TGF-*β*1-dependent activation of Smad/ERK signaling. Our study not only presents a novel mechanism underlying acquired TMZ resistance but proposes a promising strategy for therapeutic intervention in GB chemotherapy.

## Materials and methods

### Cell culture and transfection

The human GB cell line U87 was obtained from the American Type Culture Collection (Manassas, VA, USA) and U251 was purchased from the CLS Cell Lines Service GmbH (Eppelheim, Germany). Cells were maintained in DMEM with 10% (v/v) fetal bovine serum (Hyclone, Logan, UT, USA) at 37 °C in a 5% CO_2_ humidified air incubator (Thermo Scientific, Waltham, MA, USA). TMZ was obtained from Sigma (San Francisco, CA, USA) and dissolved in DMSO.

For lentiviral transduction, cells were seeded in six-well culture plate and incubated with 1 ml complete optimal medium overnight. Then medium was replaced with 1 ml mixture of complete medium with Ploybrene (5 *μ*g/ml; sc-134220, Santa Cruz Biotechnology, Santa Cruz, CA, USA). Cells were transducted by adding Control shRNA lentiviral particles (sc-108080, Santa Cruz), CTGF shRNA (h) lentiviral particles (sc-39329-v, Santa Cruz), Control lentiviral activation particles (sc-437282, Santa Cruz), CTGF lentiviral activation particles (h) (sc-400091-LAC, Santa Cruz), respectively. Medium was replaced 24 h after transfection with medium without polybrene. Transducted cells were selected for Puromycin (8 *μ*g/ml; sc-108071, Santa Cruz) resistance and screened for CTGF expression by western blot.

For siRNA transient transfection, cells were, respectively, transfected with CD44, Smad3, ERK1/2 siRNA (Supplementary Table S1) and negative Control siRNA (30 nmol/l, Sangon, Shanghai, China) using Lipofectamine 2000 (Invitrogen, Carlsbad, CA, USA) according to manufacturer’s protocol. The gene silencing efficiency was confirmed by qPCR.

### Patient tissues

Thirty-eight patients with GB who had received chemotherapy before surgery at Zhujiang Hospital (Southern Medical University, China) were included, and the histologic features of surgical resection specimens were independently examined by two neuropathologists according to the WHO criteria (Supplementary Table S2). The surgical resection specimens were then formalin fixed for further protein and RNA extractions. This study was approved by the Ethics Committee of Zhujiang Hospital and written informed consent was obtained from all patients.

### Colony formation assay

Cells were plated in 60 mm diameter culture dish at a concentration of 1 × 10^4^ cells per well. After 500 *μ*M TMZ treatment for 48 h, cells were rinsed and replaced with fresh medium, and allowed to form colonies for 7 days before being stained with Giemsa solution (Sigma-Aldrich, Darmstadt, Germany) for 15 min. After washing with PBS and air dry, the images of colonies were captured.

### Chemotherapeutic sensitivity assay

Cells were plated in 96-well plate and treated with TMZ in different concentrations. After 48 h incubation, cells were replaced with fresh medium with CCK-8 solution (v/v 10% Dojindo, Kumamoto, Japan) and incubated at 37 °C for 2 h. Then the absorbance was measured at 450 nm (reference, 620 nm) using Multiscan Go Microplate Reader (Thermo Fisher, VANTAA, Finland). Cells without TMZ treatment were set as the Control and the result was shown as cell viability ratio toward Control group.

### Flow cytometric analysis and Hoechst staining

After lentiviral transduction, cells were treated with or without TMZ and cultured for additional 48 h. After that, cells were collected and resuspended in binding buffer containing rh Annexin V-FITC for 10 min at RT. Then the cells were washed and binding buffer was added with propidium iodide (PI, 20 *μ*g/ml), and FACS analysis was performed by BD FACSCalibur (BD Biosciences Inc., Franklin, NJ, USA) according to the Annexin V-FITC Apoptosis Detection Kit (eBioscience, Waltham, MA, USA). For CD44^+^ cell proportion, anti-Human/Mouse CD44 FITC (eBioscience) was used for FACS analysis. In addition, cell apoptosis was also quantified by Hoechst 33258 (5 *μ*g/ml, Sigma-Aldrich, Darmstadt, Germany) and visualized through fluorescence microscope (Olympus, Tokyo, Japan). The images were analyzed with the Image-Pro Plus 6.0 software (Media Cybernetics, Inc., Rockville, MD, USA).

### RNA extraction and qPCR analysis, western blot analysis, immunofluorescence and immunohistochemical staining

These experiments were performed as described in Supplementary Materials and Methods.

### Enzyme-linked immunosorbent assay

After TMZ treatment, the culture supernatants of GB cells were collected, respectively, and the level of total CTGF and TGF-*β*1 was measured with CTGF ELISA Kit (OriGene Technologies, Rockville, MD, USA) or TGF-*β*1 ELISA Kit (Enzo Biochem, New York, NY, USA), respectively. The assays were carried out as recommended by the Kit protocol.

### Tumorsphere forming assay

Cell suspensions were seeded in six-well ultra-low attachment plates (Corning, Corning City, NY, USA) at a density of 5000 cells per 1 ml in Complete MammoCult Medium (StemCell Technologies, Canada). After 7 days, the images of tumorspheres were captured by a inverted microscope (Axio Observer A1, Zeiss, Oberkochen, Germany) and the number was determined using ImageJ software (National Institutes of Health, Bethesda, MD, USA). Tumorsphere with a diameter >50 *μ*M was counted in images of three fields per well in triplicate wells and the mean number of tumorspheres per field was determined. For secondary tumorsphere formation, primary tumorspheres were trypsinized, incubated and analyzed as described above.

### Tumor xenograft model

To generate murine subcutaneous tumors, 2 × 10^6^ GB cells were injected subcutaneously into the flanks of 4–6-week-old male nude mice (Laboratory Animal Center of Southern Medical University, Guangzhou, China). After tumor volume reached 50 mm^3^, the tumor-bearing mice were intraperitoneally injected for 5 days (corresponding to one cycle of TMZ treatment) per week for three cycles with 50 mg/kg TMZ in saline (final DMSO concentration 25%). Tumor volumes were calculated by the following formula: length × width^2^ × 0.5. All mice were manipulated under the protocol approved by Animal Experimental Committee of Southern Medical University.

### Statistical analysis

Results were represented as mean±S.D. for three separate experiments and analyzed by SPSS 13.0 software (SPSS Inc., Chicago, IL, USA). For experiments involving only two groups, the data were analyzed with two-Sample *t*-test assuming unequal variances, while for multiple group comparisons, statistical differences were assessed by one-way analysis of variance, followed by *post hoc* Tukey's Test. *P*<0.05 was considered as statistically significant.
