# Supplementary material for: Connective tissue growth factor promotes temozolomide resistance in glioblastoma through TGF-β1-dependent activation of Smad/ERK signaling
Source: Cell Death Dis. 2017 Jun 15;8(6):e2885–. doi: 10.1038/cddis.2017.248 (PMC5520906; doi:10.1038/cddis.2017.248)
Supplement: Supplementary Information [file cddis2017248x1.docx]

**Supplementary Materials and Methods**

**RNA extraction and qPCR analysis.** Total RNA was extracted by TRIzol Reagent (Invitrogen, USA) and the absorbance was measured at 260 nm and A260/A280. 1 μg total RNA was subjected to the synthesis of cDNA by using RevertAid First Strand cDNA Synthesis Kit (Thermo Scientific, Germany). Reactions were initiated by incubation at 65°C for 5 min, followed by 60 min at 42°C and terminated the reaction by heating at 70°C for 5 min. The cDNA performed to PCR by using the Maxima SYBR Green/ROX qPCR Master Mix (Thermo Scientific, Germany). 25 μL total reaction volume included 12.5 μL Mix (2X), 1.5 μL (10 mM) primers (Supplementary Table S3) synthesized by Sangon Biological Engineering Technology and Services Co., Ltd (Shanghai, China), 8.5 μL nuclease-free water and 0.8 μg/1μL cDNA. PCR reaction was run in Stepone Plus Real-Time PCR system (Applied Biosystems, Germany) and analyzed using Stepone software. The qPCR protocol contained initial denaturation at 95°C 10min, then 40 cycles including 95°C for 5 s denaturation, 60°C for 30 s annealing and 72°C for 30 s extension. qPCR assays were carried out in triplicate, and the specificity of the PCR products was verified with melting curve analysis. The amount of each respective amplification product was determined relative to the house-keeping gene β-actin. The fold change in gene expression relative to control was calculated by 2^-ΔΔCT^.

**Western blot analysis.** Total protein of treated cells and clinical tissue samples were extracted by Cell Lysis and Protein Extraction kit (KeyGEN Bio TECH, China) and protein concentration was measured by a BCA Protein Detection Kit (KeyGEN Bio TECH, China). 40 μg total protein was subjected to 10% SDS-poly acrylamide gel electrophoresis and transferred to PVDF membrane (Millipore Corporation, USA). The blots were blocked for 1h at RT with 5% nonfat milk (Bio-Red, USA) in Tris-buffered saline containing 0.1% Tween-20 (TBST), and probed with following primary antibodies: MDR1/ABCB1(E1Y7B) Rabbit mAb (142 kDa), MRP1/ABCC1(D708N) Rabbit mAb (173 kDa), ABCG antibody (66 kDa), MGMT Rabbit mAb (23 kDa), cleaved PARP(Asp214) (D64E10) Rabbit mAb (89 kDa), Phospho-Smad3(Ser423/425) (C25A9) Rabbit mAb (52 kDa), Phospho-p44/42 MAPK(Erk1/2) (Thr202/Tyr204) Rabbit mAb (44, 42 kDa), Phospho-p38 MAPK(Thr180/Tyr182) Rabbit mAb (43 kDa), Phospho-Akt(Ser473) Rabbit mAb (60 kDa), Phospho-SAPK/JNK (Thr183/Tyr185) Rabbit mAb (46, 54 kDa) (Cell Signaling Technology, USA), CTGF polyclonal antibody (38 kDa) (Proteintech, USA), TGF-β1(V) polyclonal antibody (25 kDa) (sc-146, Santa Cruz, USA), CD44 monoclonal antibody(82 kDa) (MAB10538, Abnova, USA) in 5% nonfat milk in TBST overnight at 4°C. Anti β-actin antibody (37 kDa) (Abcam, USA) was used as a loading control. Subsequently, the blots were washed in TBST and incubated with goat anti-rabbit or mouse IgG horseradish peroxidase-conjugated secondary antibody (Fdbio, China) for 1 h at RT. Then washed with TBST and visualized by Immobilon Western HRP Substrate (Millipore, USA). Experiments were repeated at least 3 times.

**Immunofluorescence and immunohistochemical staining.** For immunofluorescence analysis, GBM cells were seeded on glass coverslips (0.17 mm thickness, 14 mm diameter) in 6-well plate overnight, and then treated with or without TMZ for 3 d, respectively. After treatments, PBS washing and 4% paraformaldehyde fixation (30 min) were performed, followed by 0.1% Triton X-100 permeating (5 min) and 2% bovine serum albumin (BSA) blocking (30 min). Then the cells were incubated with primary antibodies diluted in 2% BSA at 4°C overnight. After 3 times PBS rinsing, appropriate fluorescent secondary antibodies were added to cell samples and incubated at 37°C in the dark for 1 h. Coverslips were mounted on slides using mounting medium (Santa Cruz, USA) contains DAPI DNA counterstain. For immunohistochemical staining, human glioma tissue sections were deparaffinized with xylene and then rehydrated through addition of ethanol. Endogenous peroxidase activity was blocked with 3% hydrogen peroxide in methanol for 10 min. Heat-induced antigen retrieval was carried out for all sections in 0.01M sodium citrate buffer, pH 6.0 at 95°C for 25 min. Anti-CTGF antibody (1:200) was incubated with sections at 4°C overnight, and then incubated with horseradish peroxidase–labeled anti-rabbit IgG for 2 h at RT. For negative controls, the primary antibody was replaced with normal goat serum. Immunofluorescent and immunohistochemical images were captured by a fluorescence microscopy (Olympus, Japan).
