# Supplementary material for: Connective tissue growth factor promotes temozolomide resistance in glioblastoma through TGF-β1-dependent activation of Smad/ERK signaling
Source: Cell Death Dis. 2017 Jun 15;8(6):e2885–. doi: 10.1038/cddis.2017.248 (PMC5520906; doi:10.1038/cddis.2017.248)
Supplement: Supplementary Tables [file cddis2017248x2.docx]

**Supplementary Table S1**

Table S1. siRNA sequences used for gene interference.

| **siRNA** | **Forward Primer (5’-3’)** | **Reverse Primer (5’-3’)** |
| --- | --- | --- |
| **CD44** | GAAAUUAGGGCCCAAUUAATT | UUAAUUGGGCCCUAAUUUCAG |
| **Smad3** | GGAGAAAUGGUGCGAGAAGtt | CUUCUCGCACCAUUUCUCCtc |
| **ERK1/2** | CCUCCAACCUGCUCAUCAA | UUGAUGAGCAGGUUGGAGG |

**Supplementary Table S2**

Table S2. Correlation between CTGF expression and clinical characteristics in 38 glioma-patients.

| **Characteristics** | **No** | **Positive of No (%)** | ***p* Value** |
| --- | --- | --- | --- |
| **Gender** |  |  |  |
| **Male** | 28 | 17 (60.7) | 0.627 |
| **Female** | 10 | 6 (60.0) |  |
| **Age, year** |  |  |  |
| **＜50** | 18 | 10 (55.6) | 0.396 |
| **≥50** | 20 | 13 (65.0) |  |
| **WHO Grade** |  |  |  |
| **I/II** | 16 | 5 (31.3) | 0.002^*^ |
| **III/IV** | 22 | 18 (81.8) |  |

**WHO: World Health Organization**

**^*^ Fisher’s exact test (*p*<0.05)**

**Supplementary Table S3**

Table S3. Primer sequences used for quantitative RT-PCR.

| **mRNA** | **Forward Primer (5’-3’)** | **Reverse Primer (5’-3’)** |
| --- | --- | --- |
| **CTGF** | GTTTGGCCCAGACCCAACTA | GGCTCTGCTTCTCTAGCCTG |
| **ALDH1** | GTCCTACTCACCGATTTGAA | CTTGTATAATAGTCGCCCCC |
| **CD44** | CACAACAACACAAATGGCTG | CAATGCCTGATCCAGAAAAA |
| **Nestin** | CGGGCTACTGAAAAGTTCC | CTGAAAGCTGAGGGAAGTC |
| **Nanog** | GAACTCTCCAACATCCTGAA | TATTCTTCGGCCAGTTGTTT |
| **TGF-β1** | CCCATGCCGCCCTCCGGGCTGC | TCAGCTGCACTTGCAGGAGC |
| **β-actin** | GACGAGGCCCAGAGCAAGAG | ATCTCCTTCTGCATCCTGTC |
